# Transactions of the Chicago Society of Physicians and Surgeons, June 22, 1874

**Published:** 1874-07-01

**Authors:** Ralph E. Starkweather


### TRANSACTIONS OF THE CHICAGO SOCIETY OF PHYSICIANS AND SURGEONS.

### REGULAR MEETING, JUNE 22, 1874.

*Reported by Ralph E. Starkweather, M.D.*

THE Society met at the Grand Pacific Hotel, the President, Dr. John Bartlett, in the chair.

Owing to the absence of Dr. Hyde, Dr. Wood was elected Secretary, *pro tempore*. Dr. H. A. Johnson received unanimous election to the membership of the Society.

Dr. Trimble read a paper on Eucalyptus. The substance of his remarks was, as he said, chiefly collected from various articles written upon the subject within the last year or two, in periodical journals; the Doctor stating that he had no practical knowledge of the effects of this medicine. Eucalyptol, the essential oil, is obtained from some of the numerous species of the eucalyptus trees of Australia, of which there are now one hundred and forty known, the E. Resinifera and E. Globulus being the varieties medicinally useful. An interesting description followed of the growth and the habits, qualities, the medicinal and mechanical properties of these trees. By distillation a water is obtained, bitter and pungent; an essential oil, and a yellow gum, aromatic in taste, shortly becoming bitter and styptic. The ashes of the wood possess the remarkable power of destroying miasmatic influences in fever-stricken districts; of absorbing ten times its weight of water from the soil, and of emitting antiseptic camphorous effluvia. A moderate dose of this liquid camphor, eucalyptol, say ten drops, may be given internally, as an antiseptic—may be used in gargles, collyria and dentrifices. It has no effect upon the eye or brain. Strong coffee is an antidote; its physiological action is very similar to that of potassium bromide. Eucalyptus may be used in treatment of dysentery, chronic diarrhœa, in diseases of the genito-urinary tract, and in remittent, intermittent and typhoid fevers. Specimens of the leaves and fluid extract were shown.

Dr. Wood—It seems important to remark that when eucalyptus is used in intermittent fever, if it is to prove useful at all, one dose generally accomplishes the desired effect: the fever does not return.

Dr. P. S. Hayes—The tincture is made stronger in alcohol than the ordinary tinctures; hence, when used with other tinctures, it precipitates; it disguises the taste of quinine.

Dr. P. S. Hayes reported a case of multilocular sero-cystic ovarian tumor. Operation—electro-puncture. Recovering. No abstract of this case is made, as it will be soon published in one of the medical journals.

Dr. Etheridge presented a report of a case of corroding ulcer of the uterus. This is a comparatively rare form of ulcer, and its diagnosis in this case is confirmed by the opinions of Drs. De Laskie Miller, Byford, and T. G. Thomas, of New York.

Patient first came under notice, July, 1871, and was fifty-nine years of age. She passed the turn of life quite suddenly in 1860. During the first twelve years after marriage she suffered from menorrhagia, at times so profusely as to prove nearly fatal. This almost wholly disappeared upon bearing children, of whom she had six. In many years of the period of child-bearing, she had stomatitis materna severely. After having had, at wide intervals, three hæmorrhages, Dr. Etheridge was called to attend the case. No examination being allowed, he ordered a vaginal injection of tanic acid, forty grains to the fluid ounce, to be used as often as necessary. Relief was afforded for four months, but the shock of the great fire in this city renewed the bleeding, but the discharges were not offensive. The only pain was a backache, a hot, burning pain, *not* lancinating. Upon examination, per vaginam, the cervix uteri was found half eaten off, the edges of the ulcer being ragged, projecting unevenly; its surface bled upon the slightest touch. Beneath the side of the os there was an indurated nodular ring around the vagina, through which the speculum always slipped with a sort of snap or jerk; uterus movable; pelvic glands and viscera normal. A solution of the pernitrate of iron temporarily checked the hæmorrhage, and afforded the longest interval of freedom from it. Other styptics were used—carbolic acid, tannin and chromic acid. Injections of the latter produced pain, often vomiting, but checked bleeding. Fifteen grains of the chromic acid in a tea-cup of warm water, seemed to be the minimum strength that was efficacious in this case. There were, at length, three months of freedom from hæmorrhage. Gradually there came on vesical trouble, and a distressing feeling in the stomach, followed by bleeding. The tissues contiguous to the ulcer now became infiltrated and indurated, probably lessening hæmorrhage; the vagina was well nigh filled with these growths; there was the usual cancerous cachexia. Morphine was used to allay pain during the latter weeks of life. No necropsy was allowed. Remedies for constitutional treatment were used, such as arsenious acid, red clover, iodine and cundurango. Prof. Schiff's treatment of uterine cancer was tried, (pancreatine solution,) with the view of dissolving the cell of the ulcer, with no satisfactory result attributable to the pancreatine.

Very particular attention was called to a point in the history above given, not alluded to in books or lectures: *The nursing sore mouth*, during lactation, *of the mother who, later in life, suffered also from malignant disease.* The cases of two other women were cited who died, after the menopause, from malignant disease, having in former years suffered from nursing sore mouth. *Is there any connection or dependence between these two conditions?* If there be, what course ought the physician to pursue with the view of preventing the development of malignancy in later years of life?

Dr. Wood stated that he knew of a family of three sisters, all of whom have had stomatitis; two of them in later life died of internal cancer; the third is now sick with the same.

Dr. Hay suggested that a table of statistics should be written, with the inquiry made by Dr. Etheridge kept in view; and it would be important, if such a relation between stomatitis materna and forms of malignant disease could be established.

Dr. Etheridge had spoken to many of the older physicians, who had given many instances similar to that of his case. The point is, whether this relationship can be established in all and sufficiently numerous cases.

Dr. Trimble reported a case of epilepsy, in a boy four years of age, probably due to injury received by railroad accident, twenty months previously Of late, the seizures had increased in frequency, severity, and mental disturbance. To eliminate the idea of worms being an exciting cause, appropriate treatment was adopted, and a few expelled. Then potassium bromide was given, four grains three times a day, but there were still as many as twenty convulsive attacks daily. Valerian was then added to the medicament to allay nervous irritability, though assafœtida was afterward substituted. Belladonna was given with the bromide. The cerebral condition soon became such that, despite the usual practice and opinion, I felt obliged to bleed, and placed four large leeches on the temples, drawing at least six fluid ounces of blood. The dose of bromide potassium was increased to eight grains. Iron and bark were given. The patient had no more seizures after the third week from the date of bleeding.

Dr. F. H. Davis, in behalf of Dr. Andrews, presented nine renal calculi, taken from one patient, by Dr. Andrews.

Dr. Walter Hay, in behalf of Dr. De Laskie Miller, exhibited a catheter which he had devised for the purpose of making medicated applications to the urethra and other tracts. It was a simple, silver catheter, with a stylet having a bulbous point, just below which a piece of medicated sponge was folded. In the stylet, near the handle, a thread was cut, upon which revolved a disk; The stylet could thus be pushed forward out of the sound to the desired distance, or the sound withdrawn upon the stylet, and the medicated sponge lying next the bulbous point, could be applied at the desired spot.

The following, upon motion of Dr. Hay, was made a by-law of the Society:

The privilege of nominating new members to the Society is restricted to the nominating by each member, of one name each year. On motion, the Society adjourned.

